# Are monitoring and evaluation systems adequate to report the programmatic coverage of HIV services among key populations in countries?

**DOI:** 10.1186/s40249-019-0570-4

**Published:** 2019-07-02

**Authors:** Jinkou Zhao, Sonia Arias Garcia, Ed Ngoksin, Jesus Maria Garcia Calleja, Chinelo Ogbuanu, Sandra Kuzmanovska, Nicholas Oliphant, David Lowrance, Nathalie Zorzi, Peter M. Hansen, Keith Sabin

**Affiliations:** 1 The Global Fund to fight AIDS, Tuberculosis and Malaria, Chemin du Pommier 40, Grand-Saconnex 1218, Geneva, Switzerland; 2 Center for Global Health, School of Public Health, Nanjing Medical University, Nanjing, China; 3 Jiangsu Provincial Center for Disease Control and Prevention, Nanjing, China; 4 Strategic Information and Evaluation Department, the Joint United Nations Programme on HIV/AIDS, Geneva, Switzerland; 5 Department of HIV/AIDS, World Health Organization, Geneva, Switzerland

## Abstract

There was no global guidance or agreement regarding when a country has an adequate system to report on the service packages among human immunodeficiency virus (HIV) key populations. This article describes an approach to categorizing the system in a country for reporting the service package among HIV key populations. The approach consists of four dimensions, namely the epidemiological significance, comprehensiveness of the service packages, geographic coverage of services, and adequacy of the monitoring system. The proposed categorization approach utilizes available information and can inform the improvement of the service delivery and monitoring systems among HIV key populations.

**Electronic supplementary material:**

The online version of this article (10.1186/s40249-019-0570-4) contains supplementary material, which is available to authorized users.

## Multilingual abstracts

Please see Additional file 1 for translations of the abstract into the five official working languages of the United Nations.

## Background

Key populations, such as sex workers, men who have sex with men (MSM), people who inject drugs (PWID), transgender people (TG) and people in prisons and other closed settings (Prisoners), are disproportionately affected by the human immunodeficiency virus (HIV) epidemic, with increased risks of acquiring and onward transmission of HIV [1]. Key populations constitute significant proportions of overall HIV burden and new infections, in all countries regardless of level of the HIV epidemic [1]. Because of punitive laws, legal and policy-related barriers, stigma and discrimination and violence, as well as other human rights violations experienced by these populations in many parts of the world, and compounded by limited availability of services, key populations are still struggling to access life-saving prevention and treatment services [1].

Reaching the majority of key populations with effective, evidence-informed and rights-based services is a prerequisite to reaching the global 90–90-90 goals, controlling the epidemic and mitigating its impact. Measuring the coverage of essential services among key populations has been a challenge for national programs, implementers, donors and multilateral organizations. Bio-behavioural surveys (BBS) among key population have been the primary source of data for coverage monitoring [2]. Bio-behavioural surveys can only be implemented with limited frequency i.e. rarely more than biennially, often less frequently, and in a limited number of sites in a country. The BBS often focuses on populations with greatest HIV burden, and/or in populations and locations where program funding has been provided. Using BBS data for national programmatic coverage monitoring is usually compromised by its limited geographic coverage, potential unrepresentativeness of data when probability sampling is not used, and poor timeliness of data given the often-prolonged periodicity of BBS implementation [2].

Routine and real-time programmatic data are increasingly generated, but remain inadequate, during program implementation, at the delivery points of prevention services, HIV testing, and support for linkages to Antiretroviral therapy (ART) initiation and retention in care for HIV-positive key populations [3]. In addition, routine laboratory and clinical management data are increasingly available, as part of the HIV care continuum. Using routine data for programmatic coverage monitoring requires an adequate monitoring system to report unduplicated utilization of essential services. Although HIV person-centred monitoring, case surveillance and data quality review guidelines [4, 5] provide general guidance and standards for monitoring systems, these references provide limited guidance specific for the monitoring of key population programs. The World Health Organization (WHO) guidelines [6, 7] list a set of comprehensive services for different key population groups and suggest a set of indicators for those services. A system for collecting the data elements for prevention indicators is not fully described. The epidemiological significance of any specific subpopulation is established by available surveillance data. Any program impact on new HIV infections and acquired immunodeficiency syndrome (AIDS)-related mortality at a national scale would require an adequate geographic coverage of a comprehensive service package maintained over time. Scattered program sites in a few places, in many cases, in the national or provincial capitals only, cannot achieve any measurable impact at national level in reducing HIV incidence and/or AIDS mortality. Furthermore, there is no consensus in defining when the geographic or affected population coverage, periodicity of data collection, and monitoring system attributes are sufficient to demonstrate such impact.

The present article discusses critical dimensions and attributes of an effective monitoring system, and proposes a practical categorization matrix. Using standard criteria across four dimensions, the authors propose a simple system to determine the adequacy and utility of reported programmatic coverage of HIV services among key population in 55 low- and middle-income countries with existing data systems.

## Main text

### Key population groups of epidemiological significance

A key population is a group defined by behaviours that place members at increased risk of contracting or transmitting HIV. The epidemiological significance of the most common key populations, namely sex workers of any gender, gay men and other men who have sex with men, people who inject drugs, trans women, and prisoners is seen through the disproportionate burden of disease among these populations. Inclusion of other, locally defined key subpopulations is beyond the scope of this review because of a lack of universality [7, 8].

A precursor step to evaluating the monitoring system for key populations programming is to determine the relative epidemiological significance of a key population to assure that all of the most important populations are included. This is achieved by examining the contribution of each group to HIV acquisition, onward transmission, and AIDS-related morbidity and mortality. The estimated number of people living with HIV (PLHIV) by key population groups offers a good proxy for defining epidemiological significance. Such estimates are only consistently available in a dozen or so Asian countries and a few African countries where the AIDS Epidemic Model [9] or other dynamic estimation models [10] are applied to derive national HIV estimates. Any efforts to derive such estimates more broadly would require, among other data, higher quality population size estimates and HIV prevalence data parsed by group and time.

The overall size of a key population is another proxy for potential epidemiological significance. Population size estimates are now available from more than 120 low- and middle-income countries but with dramatically varied quality. Until recently, there has not been a centralized repository for such data. Global public health agencies, including the Global Fund, WHO and the United Nations Joint Programme on HIV/AIDS (UNAIDS) systematically assessed the quality of population size estimates and categorized the countries into, (1) nationally adequate, (2) nationally inadequate but locally adequate in selected sites, (3) documented estimates but inadequate methods, (4) undocumented or untimely, and (5) no data [11].

A third proxy for epidemiological significance is HIV prevalence. Prevalence of HIV among key populations is routinely collected and captured through UNAIDS Global AIDS Monitoring (GAM) reporting, where the quality is reviewed by the national authority and UNAIDS regional and country offices [11]. Availability to the national program of HIV prevalence data among key populations is considered ‘good’ if data for at least two key population groups are reported through GAM.

Assessment of the availability of all the above information used to define the epidemiological significance indicates that HIV prevalence is the most commonly available data. The two key population groups with the highest HIV prevalence ever reported are selected as the most epidemiologically significant groups.

### Comprehensiveness of service package (X)

WHO and United Nations partners define the comprehensive package of services for all key populations with specific considerations for each of the key population groups, as shown in Tables 1 [6].

**Table 1:** The services for each of key populations, as listed in WHO guidelines [5]

Key population groups	Essential health sector intervention	Essential strategies for an enabling environment	Specific considerations
Sex workers	• Comprehensive condom and lubricant programming • Harm reduction interventions for substance use, in particular needle and syringe programs (NSP) and opioid substitution therapy (OST) • Behavioral interventions • HIV testing and counselling • HIV treatment and care • Prevention and management of co-infections and other comorbidities, including viral hepatitis, TB and mental health conditions • Sexual and reproductive health interventions	• Supportive legislation, policy and financial commitment, including decriminalization of behaviors of key populations • Addressing stigma and discrimination • Community empowerment • Addressing violence against people from key populations.	• Correct and consistent use of condoms and condom-compatible lubricants is recommended for sex workers and their clients • Female condoms for female sex workers (FSW), particularly FSW who inject drugs, for power imbalance during condom negotiation
Men who have sex with men			• Condoms and condom-compatible lubricants are recommended for anal sex • Adequate provision of lubricants needs to be emphasized
People who inject drugs			• Immediate implementation of NSP and OST • Condom programming is an essential component of the comprehensive harm reduction package for people who inject drugs and their sexual partners
Transgender people			• Condoms and condom-compatible lubricants are recommended for anal sex • Adequate provision of lubricants for transgender women and transgender men who have sex with men needs emphasis
People in prisons and other closed settings			• Prevention of HIV transmission through medical and dental services • Prevention of transmission of HIV and other bloodborne diseases through tattooing, piercing and other forms of skin penetration • Protecting staff from occupational hazards • Condom and lubricant distribution programs in prisons and other closed settings, without quantity restriction, with anonymity and in an easily accessible manner

Several real-world issues often limit the implementation of all components of the comprehensive package of services. Such factors range from political will, legal and policy environment, to local capacity and resources. Tracking coverage of key populations receiving all the items in the comprehensive package is simply unrealistic, as not all items are directly provided to the individuals at one time, even within, for example, an annual implementation period. During program implementation, no individual key population member may need all items from the overall package at the same time and with the same frequency. For example, people who inject drugs only may be in need of certain interventions in the package of harm reduction services (such as Needle and Syringe Program [NSP], Opioid Substitution Therapy [OST], HIV Testing Services [HTS], etc.) and might not need sexually transmitted infections (STI) services and HIV treatment and care. In addition, not all service providers offer the full list of interventions included in the package. For instance, a specific community-based service provider may not provide these services themselves but may refer individuals to health facilities for diagnosis and treatment of sexually transmitted diseases. Programs often lack data systems to track individuals across different service providers.

The list of comprehensive services in the WHO guidelines [6] include essential strategies for an enabling environment, such as those to decrease stigma and discrimination. These are not included in the present assessment. Thus, only the essential items that are recommended to be provided directly to individuals are included, as those services for an enabling environment require a different measurement system.

For PWID, essential harm reduction interventions are NSP and OST. Using all available documents, including GAM reporting, the Global Fund grant reports and a special assessment of key population services commissioned by the Global Fund [12], we found that all countries reporting OST services also reported NSP services.

The following services are therefore included in the comprehensiveness of service package assessment for all key population groups.

- Comprehensive condom and lubricant programming
- Behavioural interventions
- HIV testing services
- HIV treatment and care
- Any of the following,
  - Sexual and reproductive health interventions: Sexually transmitted infection prevention, screening and treatment
  - Hepatitis prevention and management, co-infections
  - TB prevention and management, co-infections

Additionally, NSP is included for PWID.

The comprehensiveness of service packages was assessed based on whether the design of the service package at country level was aligned with the technical guidelines; and whether the designed package of services was implemented accordingly. In order to categorize countries, each of the key population groups was assigned a score against each of the services, based on the following categories,

1. No data/ Not assessed / Not observed
2. Designed but no evidence of implementation
3. Designed and evidence of implementation

Individual scores were added up, with a total ranging from 0 to 10 for each key population. The sum score for PWID was weighted to adjust it to the scale of 0 to 10, because of the additional package element (NSP).

### Geographic coverage of services (Y)

Effective epidemic control will require near universal health coverage of HIV prevention and treatment services among key populations. Universal health coverage [13] is defined as ‘ensuring that all people have access to needed health services (including prevention, promotion, treatment, rehabilitation and palliation) of sufficient quality to be effective while also ensuring that the use of these services does not expose the user to financial hardship.’ Based on this definition, routine health services shall be universally accessible, irrespective of geography and groups. All services essential for preventing and treating HIV among key populations shall be integrated into routine health services wherever there is a health service site. However, it is typical for key populations that services are delivered by different providers, with the care cascade provided in a fragmented manner in many countries. Many services are stand-alone and available only in big cities where key populations congregate, and the remaining population may be more tolerant. Many services are offered by community groups and may not be incorporated into the national reporting system.

Service availability and access are often compromised due to contextual factors, such as stigma associated with certain behaviours, sexual orientation or gender identity. Discrimination against people living with HIV and criminalization of certain behaviours exacerbates stigma and further contributes to poor access and/or uptake of services. This is particularly profound in areas where people are intolerant of behaviours which are different from their personal beliefs. Rural areas, closed communities, geographically limited areas, and fragile settings, are among such examples. Stand-alone service provision in these areas puts key population clients at unnecessary risks. Key population clients may rather access the services at general health service sites by not disclosing risk behaviours, sexual orientation or gender identity.

When assessing the geographic coverage of the services for key populations, the focus is therefore on the areas where large numbers of key populations congregate, and services are available and used. However, for the program to have a population-level impact, the services cannot be geographically limited to very few big cities, such as the capital city only, but should go beyond the capital and big cities to reach other subnational geographic divisions.

The following scoring system was used to score the service availability in geographic areas,

1. No reported services available in the country
2. Subnational level: when services are available and in a few first-order administrative divisions (such as provinces, regions or states) only
3. National level: when all the first-order administrative divisions (such as provinces, regions or states) of the country provide services.

We used the following data sources for the assessment,

- 2017 UNAIDS Global AIDS Monitoring (GAM) [14]: ‘Number of administrative areas with service provision sites’ by key population, compared with the number of administrative areas in the country.
- 2017 PEPFAR KP-PREV data [15]: The number of SNU1 (first level of subnational unit) where intervention service results were reported for all key populations in any quarter of 2017. As the data were not disaggregated by key population groups, the same value was used across key population groups.
- Remaining countries: For countries where none of the above were available, data were abstracted from the Global Fund grant progress update in 2017. Additionally, the information was abstracted from the desk review reports produced by APMG Health [12].

### Adequacy of monitoring systems (Z)

A well-functioning monitoring system is indispensable for tracking the progress of the program while keeping the service recipients safe. Confidentiality is an important concern when recording data about key populations. Given the ongoing nature of major services for key populations, individuals may access services repeatedly and among different service providers. Lack of personal identifiers in the recording systems usually result in duplicated recording and reporting of the programmatic results. Assigning a unique identification code (UIC) to each individual, instead of using their names, is a good method to protect confidentiality and privacy of individuals captured in the program records, while ensuring the unduplicated reporting of programmatic results. Different providers, often financed by different funders, may have different UICs. Ideally, different UIC systems need to be harmonized, and implemented consistently over time and across service providers, in order to enable the reporting of national service coverage.

Countries are in different stages of development of monitoring systems. The adequacy of the systems was assessed against whether key population individuals reached with services were counted and reported by service contacts or as individual clients, with scores proposed as following,

1. No data or evidence of existence of a monitoring system
2. Monitoring contacts, which disallow de-duplicated reporting
3. Partially using UIC, which disallows de-duplicated reporting. This includes scenarios where UICs are used in some regions of the country or different UICs are used in the country but not harmonized.
4. Nationally using UIC, which allows for de-duplicated reporting. This includes the scenario where different UICs are used but harmonized.

### Consolidating four dimensions

After the individual scores for each dimension were calculated for each of the selected key populations, the scores were adjusted with differing weights, to account for the relative importance of each dimension, and then summed up for all the key populations in each country, as described in the following formula:

$$ \mathrm{Sum}\ \mathrm{Score}\left(\mathrm{by}\ \mathrm{key}\ \mathrm{population}\right)=\left(\mathrm{X}\ast 3\right)/10+\left(\mathrm{Y}\ast 3\right)/2+\mathrm{Z} $$

After the adjustment, the possible Sum Score for each key population ranged from 0 to 9, described below in Table 2.

**Table 2:** Illustration of country specific scores

Country	Key population 1	Key population 2	TOTAL
A	A1	A2	A1 + A2
B	B1	B2	B1 + B2
C	C1	C2	C1 + C2
D	D1	D2	D1 + D2
…			

With the calculation above, the final sum score for a given country with two key population groups could range between 0 and 18. To categorize the countries on their adequacy to report the programmatic coverage of services among key populations, the following ranges are proposed:

- Unable to report in the next 3 years: The sum score is less than 9, 50% of the sum score, or the Adequacy of Monitoring System had a score of 0 or 1. These are countries that need significant improvements before they can report.
- Potentially able to report in the next 2–3 years: The sum score is between 9 and 13.4, and the Adequacy of Monitoring System has a score of 2 or 3. Countries could potentially report on coverage of services among key populations when certain improvements are made.
- Able to report now: The sum score is equal to or greater than 13.5 and the Adequacy of Monitoring System has a score of 2 or 3. These are countries that are able to report even though constant improvements are still required.

The categorization shall be regularly updated, when new data and information become available through the Global Fund grant progress updates, GAM reporting, the President’s Emergency Plan for AIDS Relief (PEPFAR) reporting and reports from other partners.

**Fig. 1:**
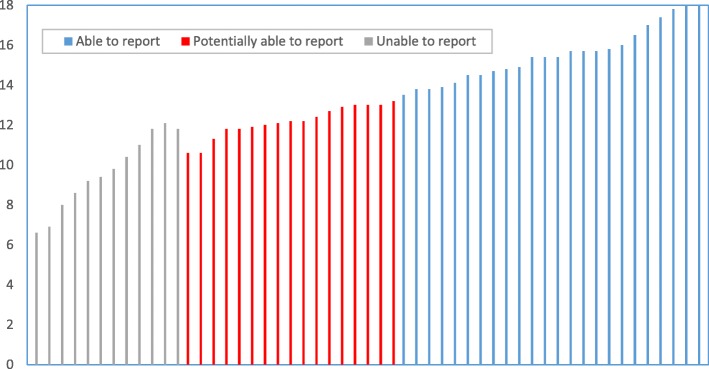
Categorization of countries on the capacity to report on comprehensive package of services among key populations

Based on the results of joint assessments of available size estimates of key populations, 53 countries were classified as having nationally adequate size estimation for at least two key population groups as of 2017. Applying the criteria proposed above, these 55 countries were categorized as depicted in Fig. 1. Out of 53 countries, 24 were ‘able to report now’, while 12 were ‘unable to report in the next 3 years’. Eight countries with a sum score above 9 were classified as ‘unable to report in the next 3 years’ because their scores for Adequacy of Monitoring System were either 0 or 1.

### Data quality and limitations

This assessment does not address the data quality of individual estimates or numbers reported; it was an assessment of the systems that collect the data. Data used for the current assessment are all secondary, reported by implementers or national programs to UNAIDS, WHO, the Global Fund or PEPFAR, and subject to quality assurance, including timeliness and completeness within the respective reporting systems and consistent with internal guidelines of the respective implementers and donors [13–16]. For example, the Global Fund grants-supported results are verified by Local Fund Agents, and externally audited by commissioned providers; and PEPFAR results are verified and audited on an ongoing basis by the implementing partners and external providers. For GAM reporting, prevalence and size estimation data are assessed at different stages by in-country and regional authorities and by UNAIDS and WHO staff in Geneva. Although this assessment did not include the services for an enabling environment, the exclusion of such services should not be interpreted as exclusion from budgeting and implementation in the respective countries.

Several issues were noted in using HIV prevalence among key populations to define their epidemiological significance. Firstly, the limited number of sites where the HIV prevalence data were collected was often non-representative of the country, even inadequate to perform extrapolation exercises. Secondly, some sampling methods used were not probability-based, nor even quasi-probability, such as respondent-driven sampling or time-location sampling. This created challenges for assessing the representativeness of the survey sample for the population at every survey site. Thirdly, there were scenarios where HIV prevalence was high and estimated population size was small for a given key population, which may not have contributed significantly to the overall HIV burden among all key populations. Low overall HIV burden may have affected resource allocation but it should not absolve the national response from making services available for affected populations, particularly considering the emerging global commitment to universal health coverage.

Quality assurance within the monitoring systems was not reviewed in this article. It is another essential system attribute to be considered in future assessments, which will require augmented reporting of such information.

## Conclusions

Adequate programmatic service coverage levels among key populations affected by the HIV epidemic is a prerequisite to achieving overall HIV epidemic control as well as control among key population communities. The package of services should be designed and implemented in alignment with the most up-to-date technical guidelines and tailored to the local epidemiologic and environmental contexts. The assessment results described categorization of countries’ monitoring systems based on their adequacy in reporting programmatic service coverage among key populations. The categorization should provide insight to countries and partners in describing the extent to which programmatic coverage data reported by countries can be used for cascade monitoring purposes. The categorization can identify gaps in each of dimensions to facilitate incremental improvements in both monitoring systems and, resultantly, in service coverage and quality.

It is important to bear in mind however, that in order to apply this categorization, the country must have a ‘nationally adequate’ [10] size estimation for its key populations as a reliable denominator. The categorization proposed in this article is also intended to facilitate countries conducting key population size estimation activities and improving their quality, not only as a critical denominator for the programmatic coverage calculation, but in understanding the magnitude of the service and quality gaps to inform planning and advocacy efforts. The inclusion of particular services in this categorization does not mean that only these services should be implemented. The services defined in the WHO guidelines for an enabling environment are equally important and should be properly budgeted for and implemented.

## Additional file

Additional file 1: Multilingual abstracts in the five official working languages of the United Nations. (PDF 355 kb)

## Data Availability

The data are partially available at the UNAIDS AIDSInfo at the link below. Other data which belong to the Global Fund or PEPFAR, may be available upon request but subject to respective internal approval. http://www.aidsinfoonline.org/kpatlas/#/home

## Abbreviations

AIDS: Acquired immunodeficency syndrome
APMG: AIDS project management group
BBS: Bio-behavioral survey
GAM: Global AIDS monitoring
HIV: Human immunodeficiency virus
HTS: HIV testing services
KP-PREV: Key populations prevention
MSM: Men who have sex with men
NSP: Needle and syringe program
OST: Opioid substitution therapy
PEPFAR: The U.S. President’s Emergency Plan for AIDS Relief
PLHIV: People living with HIV
PWID: People who inject drugs
SNU: Subnational unit
STI: Sexually transmitted infections
TB: Tuberculosis
TG: Transgender people
UIC: Unique identification code
UN: The United Nations
UNAIDS: The Joint United Nations Programme on HIV/AIDS
WHO: World Health Organization
