# Supplementary material for: Are monitoring and evaluation systems adequate to report the programmatic coverage of HIV services among key populations in countries?
Source: Infect Dis Poverty. 2019 Jul 2;8:58. doi: 10.1186/s40249-019-0570-4 (PMC6604138; doi:10.1186/s40249-019-0570-4)
Supplement: Supplementary file 1 — Multilingual abstracts in the five official working languages of the United Nations. (PDF 355 kb) [file 40249_2019_570_MOESM1_ESM.pdf]

Translation of the abstract into the five official working languages of the United Nations

هل أنظمة المراقبة والتقييم كافية للإبلاغ عن التغطية البرنامجية لخدمات الوقاية من فيروس نقص المناعة البشري بين المصابين؟

جين كو تشاو، سونيا أرياس جارسيا، إد نغوكسان جيسوس ماريا جارسيا كاليخا، تشينيلو أوغبوانو، ساندرا كوزمانوفسكا، نيكولاس أوليفانت، ديفيد لورينس، ناتالي زورزي، بيتر إم هانسن، وكيث سابين

الملخص
لم يكن هناك إرشاد عالمي أو اتفاق فيما يتعلق بامتلاك بلد ما نظامًا كافيًا للإبلاغ عن الخدمات المقدمة للمصابين بفيروس نقص المناعة (HIV).
يصف هذا المقال طريقة تصنف النظام في بلد ما للإبلاغ عن الخدمات المقدمة للمصابين بفيروس نقص المناعة البشرية. تتألف هذه الطريقة من أربعة محاور: الأهمية الوبائية، وشمولية الخدمات المقدمة، والمنطقة الجغرافية التي تغطيها الخدمات، وكفاية نظام المراقبة.
تستخدم طريقة التصنيف المقترحة المعلومات المتوفرة وتبلغ عن أي تحسينات في توصيل الخدمات وأنظمة المراقبة للمصابين بفيروس نقص المناعة البشرية.

Translated from English version into Arabic by Rahaf Alkharouf, Revised by Mohamed Shawkat, through

TRANSLATORS
WITHOUT BORDERS

国家层面的督导和评估系统是否足以报告艾滋病高危人群服务的覆盖面？

Jin-Kou Zhao, Sonia Arias Garcia, Ed Ngoksin, Jesus Maria Garcia Calleja, Chinelo Ogbuanu, Sandra Kuzmanovska, Nicholas Oliphant, David Lowrance, Nathalie Zorzi, Peter M. Hansen amd Keith Sabin

摘要
在报告艾滋病高危人群服务覆盖面时，一个国家的报告系统是否充分，目前尚无全球指南或一致意见。本文介绍了一种方法，对国家层面的报告系统进行分类，该方法有四个方面，即流行病学意义、服务包的完整性、服务的地理覆盖范围以及监测系统的充分性。结果显示，提议的分类方法可以利用现有信息，来改进针对艾滋病高危人群中的服务及其监测系统。

Translated from English version into Chinese by Jin-Kou Zhao

Les systèmes de suivi et d'évaluation permettent-ils de rendre compte de la couverture programmatique des services liés au VIH parmi les populations clés?

Jin-Kou Zhao, Sonia Arias Garcia, Ed Ngoksin, Jesus Maria Garcia Calleja, Chinelo Ogbuanu, Sandra Kuzmanovska, Nicholas Oliphant, David Lowrance, Nathalie Zorzi, Peter M. Hansen et Keith Sabin

Résumé

Il n’y a pas d'orientations ni d'accords internationaux permettant de dire à quel moment un pays dispose d’un système adapté pour rendre compte de l'ensemble des services sur les populations clés atteintes du virus de l’immunodéficience humaine (VIH).

Cet article propose une catégorisation des systèmes de suivi des services apportés aux populations clés atteintes du VIH dans un pays donné. Cette approche repose sur quatre dimensions, à savoir l'importance épidémiologique, l'exhaustivité des ensembles de services, la couverture géographique des services et l'adéquation du système de surveillance.

L'approche proposée utilise les informations disponibles et peut contribuer à l'amélioration des systèmes de prestation de services et de surveillance des populations clés atteintes du VIH.

Translated from English version into French by Ingrid Clément, Revised by Eric Cote, through

Являются ли системы мониторинга и оценки достаточными для предоставления отчетности по программному охвату услугами для ВИЧ среди ключевых групп населения?

Джин-Коу Чжао, Соня Ариас Гарсия, Эд Нгоксин, Хесус Мария Гарсия Каллея, Чинело Огбуану, Сандра Кузмановская, Николас Олифант, Дэвид Лоуранс, Натали Зорзи, Питер М. Хансен и Кит Сабин

Аннотация

Не существует глобального руководства или соглашения относительно того, когда страна имеет надлежащую систему предоставления отчетов по пакетам услуг для ключевых групп населения с вирусом иммунодефицита человека (ВИЧ).

В статье описывается подход к разделению данной системы на категории в стране для предоставления отчетов по пакетам услуг для ключевых групп населения с ВИЧ-инфекцией. Данный подход состоит из четырех аспектов, а именно, значимость с эпидемиологической точки зрения, полнота пакетов услуг, географический охват предоставления услуг и достаточность системы мониторинга.

Предложенный метод классификации основывается на доступной информации и может информировать об улучшении предоставления услуг и систем мониторинга ключевых групп населения с ВИЧ-инфекцией.

Translated from English version into Russian by Veronika Demeshchyk, Revised by Anna Kukharchuk, through

¿Son adecuados los sistemas de supervisión y evaluación para informar sobre la cobertura de los programas de los servicios de atención del VIH entre las poblaciones clave?

Jin-Kou Zhao, Sonia Arias Garcia, Ed Ngoksin, Jesus Maria Garcia Calleja, Chinelo Ogbuanu, Sandra Kuzmanovska, Nicholas Oliphant, David Lowrance, Nathalie Zorzi, Peter M. Hansen y Keith Sabin

Resumen

No hubo ninguna orientación mundial ni un acuerdo sobre cuándo un país cuenta con un sistema adecuado para informar sobre los paquetes de servicios entre las poblaciones clave infectadas con el virus de la inmunodeficiencia humana (VIH).

El presente artículo describe un enfoque para categorizar el sistema de un país para informar sobre el paquete de servicios entre las poblaciones clave infectadas con el VIH. El enfoque consta de cuatro dimensiones, a saber, la importancia epidemiológica, la exhaustividad de los paquetes de servicios, la cobertura geográfica de los servicios y la adecuación del sistema de supervisión.

El enfoque de categorización propuesto utiliza la información disponible y puede informar sobre la mejora de la prestación de servicios y los sistemas de supervisión entre las poblaciones clave infectadas con el VIH.

Translated from English version into Spanish by Mayra León, Revised by Patricia Cassoni, through
